# Traditional and modern practitioners' perceptions on traditional medicine use in reproductive health among the Khoisan of Zimbabwe

**DOI:** 10.3389/frph.2026.1784279

**Published:** 2026-06-19

**Authors:** Nicholas Mudonhi, Boikhutso Tlou, Wilfred Njabulo Nunu

**Affiliations:** 1 Department of Public Health, School of Nursing and Public Health, College of Health Sciences, University of KwaZulu-Natal, Durban, South Africa; 2 Department of Environmental Health, Faculty of Environmental Sciences, National University of Science and Technology, Bulawayo, Zimbabwe; 3 Department of Environmental Health, School of Public Health, Faculty of Health Sciences, University of Botswana, Gaborone, Botswana; 4 Department of Environmental Health, Bulawayo Polytechnic College, Bulawayo, Zimbabwe

**Keywords:** Khoisan, modern practitioners, reproductive health, traditional medicine, traditional practitioners, Zimbabwe

## Abstract

**Background:**

Traditional health systems often serve as the first point of contact for reproductive health issues in rural areas of Zimbabwe. Khoisan people rarely use contemporary health facilities for therapy, as herbal treatments are an essential component of their culture.

**Aim:**

This study aimed to explore the Perceptions of Traditional and modern practitioners regarding the use of traditional medicine in reproductive health among the Khoisan of Zimbabwe.

**Methods:**

A phenomenological study was conducted withto fifteen purposively selected health professionals, which includied nurses, doctors, and environmental health technicians, as well as snowballed twenty six traditional health practitioners, who responded to unstructured interviews openly on sensitive reproductive topics. Data were collected using a tape recorder, transcribed, and then thematically analysed using MAXQDA.

**Results:**

The collected and evaluated data revealed seven key themes and twenty-three subcategory themes. The key themes associated with the use of traditional medicine in reproductive health among the Khoisan people of Bulilima and Tsholotsho Districts were as follows: Types of traditional medicines, Administration and Preparation Techniques, Reproductive Practices, Knowledge Transmission, Perceived Efficacy and Safety, Research and Education. *Colophospermum mopane* roots, whole plants of *Pouzolzia hypoleuca*, water snake/fish, soot from old, thatched kitchens, and donkey placentas were used to shorten labor and ease delivery. *Salvadora persica L*, *Ximenia caffra*, *Solanum campylacanthum*, *Sclerocarya caffra*, and *Drimia sanguinea* have been used to treat gonorrhea.

**Conclusion:**

The Khoisan people of Zimbabwe rely heavily on traditional medicine to treat sexually transmitted diseases, control pregnancy, and engage in sexual behaviors. The administration of these herbs vary from topical, oral and steam baths in dealing with health outcomes. An ethnographic study should be conducted to determine the hidden constituents that are not limited to common herbs

## 1 Background

The WHO's 2025–2034 policy emphasizes the importance of validating traditional medicines in order to enhance health outcomes and integrate them into the national health system (1). Proper use of traditional medicine makes a substantial contribution to a number of UN goals, including SDG 3 (excellent health and well-being), SDG 8 (economic growth), SDG 12 (sustainable consumption), and SDG 15 (life on land) (1, 2). Literature reveals that traditional health systems often serve as the primary contact for reproductive health issues in rural areas of Zimbabwe (3–5, 64). It has been noted that local populations in African regions, notably the Khoisan of Zimbabwe, South Africa, Namibia and Botswana rely heavily on traditional medicine for reproductive health care (6–8). In addition, Khoisan people rarely use contemporary health facilities for therapy because herbal treatments are an essential component of their culture (8). Several studies have observed that, recently and over the past few decades, traditional medicines have provided not only phytochemical and biological interventions but also a holistic, broader-spectrum approach to health that includes ancestral and spiritual elements (9–11). This is further supported by a study conducted by Low in 2004, which indicates that the Khoisan people use and understand traditional medicine as part of their culture and family structures (8).

The Khoisan are a mixture of the San and Khoi people and are believed to be the founders of most parts of the Southern African Region, who preserve their culture (7). Even today, they subscribe to a nomadic way of living and possess a vast understanding of nature, ancestral spirits, and communal rituals that inform their use of ethnomedicine and therapeutic practices (8, 12). These traditional activities are not only healing, but also promote social connections and identity preservation. Herbal health practices of local people are not only healing, but also promote social connection and identity preservation (13). Several authors in Zimbabwe have highlighted that ethnomedicine is frequently utilised in rural and marginalized cultures with limited access to hospitals compared to urban settings (13–15). In recent years, the Zimbabwe National Traditional Healers Association (ZINATHA), has played a crucial role in safeguarding public safety by crafting policies that govern the utilisation of traditional medicines in managing different health ailments (5, 16, 17). Despite the existence of this association since 1980s, traditional medicine remains understudied, particularly in terms of sexual habits, STIs, and maternal health among indigenous communities, such as the Khoisan (13, 17). It has been observed that many choose not to consult modern health systems for fear of discovering their HIV status and other health issues. Geographical, cultural, and systemic restrictions have been recognised as contributing to limited access to reproductive health treatments (16).

Research conducted in Zimbabwe noted a complex relationship between cultural beliefs about ethnomedicine use and Western healthcare professionals' pessimism about the safety and efficacy of these indigenous medicines (15). Therefore, there is a need to implement merging advanced laboratory techniques that honour traditional systems to enhance the health of the entire population and meet SDGs 3, 8, 12 and 15 (1, 18). Due to cultural erosion and cross-marriages, the Khoisan of Zimbabwe is regarded as a tribe with low population demographics. However, despite having less accessible information online on their traditional practices than other ethnic groups, they provide a unique framework for studying the integration and divergence of traditional and modern reproductive health techniques (19). Indigenous peoples' therapeutic practices are influenced by their harmony with nature as well as social beliefs, values, and norms, and offer valuable insights regarding adaptation, cultural continuity, and health-seeking behaviours (8). This study aims to address a significant research gap in the literature by employing a qualitative approach to understand traditional and modern practitioners perceptions on reproductive health issues among the Khoisan. The specific objectives is to document traditional medicines patterns, determine practitioner narratives on utilisation, and assess the feasibility of integrative healthcare models that respect both systems.

## 2 Methods

### 2.1 Study area

**Figure 1:**
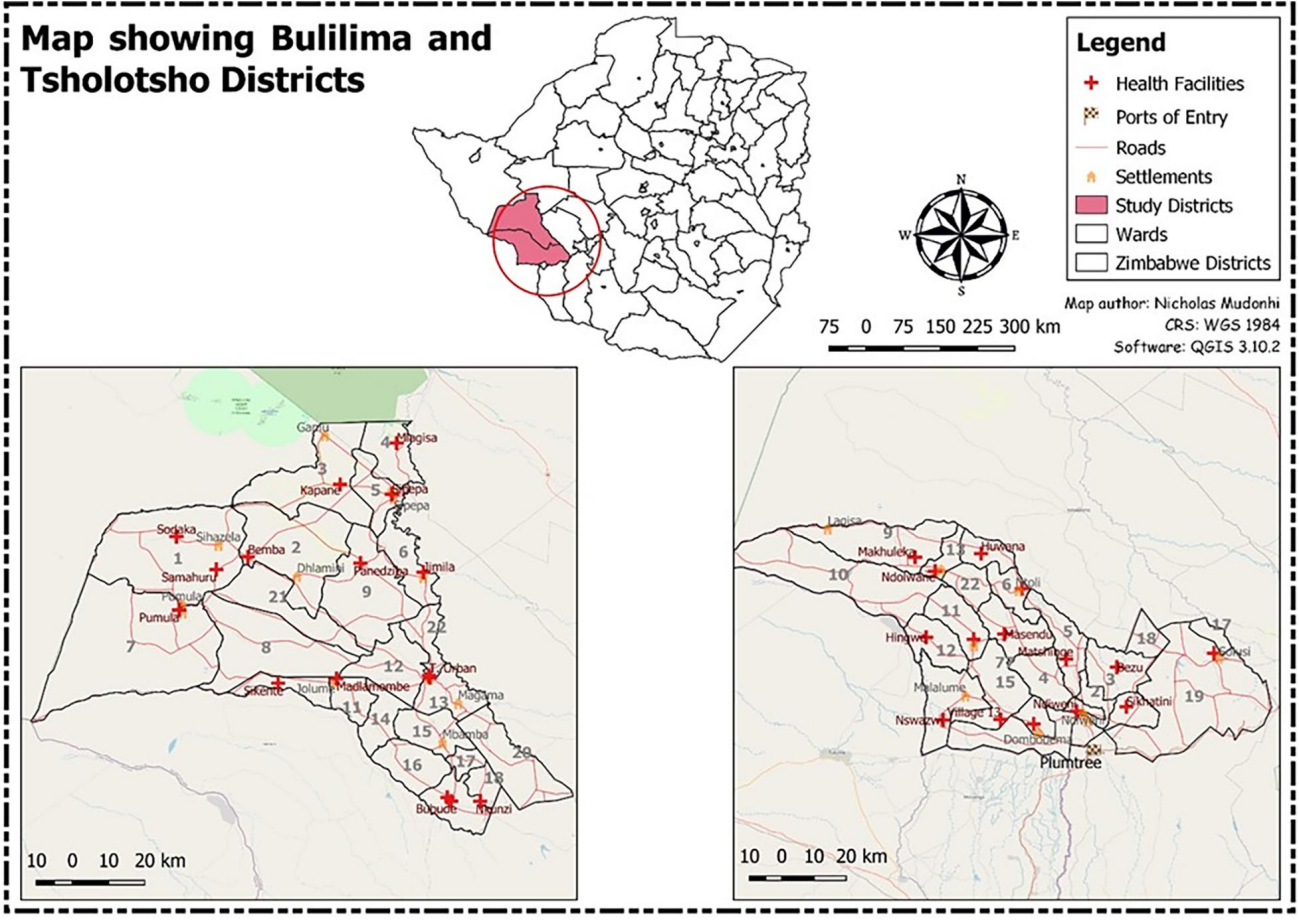
Map showing Bulilima and Tsholotsho districts.

The research was conducted in the Matabeleland North and South Provinces, specifically in the Tsholotsho and Bulilima Districts. These districts were intentionally selected because of their strong, distinctive cultures and unique status as Zimbabwe's only two districts with villages inhabited by a substantial number of Khoisan. In the Zimbabwean context, the Khoisan are a mixture of the San and Khoikhoi people who are noted to be the oldest and most native tribes. Khoisan societies used to speak the Tshwao language, but because of cultural erosion, the predominant languages are now Kalanga and Ndebele. Tshwao are well-known for their strong methods of conserving their culture using traditional knowledge systems for healing and optimal health. The dominant languages spoken in these districts are Tshwao, Kalanga, and Ndebele, with Tshwao currently being known and communicated by a few elders in the community and thus likely to be eroded. The study was conducted at two referral centers in these districts, Plumtree and Tsholotsho Hospitals, where health professionals are located. In addition, the research also considered acquiring information from clinics within a ten-kilometre radius of Khoisan village, including the Makhulela Clinic in Plumtree, Mpilo, Sikente Clinics, and Pumula Mission Hospital in Tsholotsho. In Bulima District, the study was conducted in the Newline and Thwaithwai/Sabasi areas, while in Tsholotsho, it was carried out along the Mtshina, Butabubili, and Gariya lines. A map of the study area is presented in Figure 1.

### 2.2 Study design

A phenomenological research design was employed to explore stakeholders' lived experiences and perspectives on the use of traditional medicines in reproductive health. These experts consented to the study, and data were collected using interview questions, which were recorded digitally and in cases where recording was declined, detailed handwritten notes were taken instead. The collected data were used to generate themes and subcategories, these emergent themes were subsequently shared with relevant stakeholders to deepen understanding of the specific herbs, preparations, and traditional formulations used in reproductive health. This design enabled an in-depth exploration of culturally embedded practices, allowing for richer insights into traditional reproductive health interventions.

### 2.3 Target population

The research included traditional practitioners, such as herbalists, bone setters, diviners, traditional midwives, and faith healers, as well as health practitioners, including general nurses, nurse aides, medical doctors, and environmental health technicians, who dealt with reproductive health issues. Although general nurses, nurse aides, medical doctors, and environmental health technicians are not formally trained as traditional medicine experts, they were purposefully selected due to their frequent interaction with khoisan using traditional therapies for reproductive health concerns. These practitioners work on the front lines of healthcare delivery and are thus well positioned to provide educated opinions on the patterns, perceived effects, and clinical consequences of traditional medicine use.

### 2.4 Sampling and sample size

Tsholotsho and Bulilima districts were purposively selected because they are the only districts in Zimbabwe with a higher population of Khoisan people. Snowball sampling was utilised to recruit 26 traditional practitioners, and data collection continued until saturation was reached, as evidenced by repeated responses and the absence of new themes, practices, or herbal remedies in subsequent interviews. This technique was employed because most traditional practitioners are located in hard-to-reach areas where accessibility without prior knowledge of their whereabouts might be challenging and their dwellings are scattered across the village. Furthermore, this technique reduces both time and cost as it was easy to locate due to referrals from others. In addition, the snowball technique was used because it increases participant recruitment by using existing professional and social circles. When an initial participant recommends others in their network, the suggested individual are more likely to participate since they trust and know the referrer. Peer-to-peer endorsement strengthens study trustworthiness, reduces reluctance, and enhances response rates, especially for sensitive subject matters in reproductive health among khoisan (20). Purposive sampling was used to recruit 15 health practitioners (doctors, nurses, and environmental health practitioners), as they possess firsthand and in-depth knowledge and expertise of reproductive health issues (21). In addition, convectional health proffessionals practice health education to San community making them ideal to have information regarding their reproductive health and use of traditional medicine.

### 2.5 Data collection tools

Qualitative data were gathered from sunrise to sunset in June, July, and August 2025, with the aid of in-depth 45–60 min interviews with key informants (traditional and health practitioners). Interview guides were initially created in English and then translated into Ndebele and Kalanga, the two main languages spoken and understood by Khoisan within the district. Traditional practitioners were interviewed in their local languages that is isiNdebele and Kalanga by the researcher and interpreter while Health Practitioners were interviwed in english. The interview guide for traditional practitioners comprised 14 questions on demographic characteristics, traditional healing practices, reasons for using TM, safety, and the challenges of utilising ethnomedicine in reproductive health. The interview guides for health practitioners included seven questions on their role in reproductive health, knowledge of TM utilised in sexual practices, STIs, and pregnancy-related issues, and how it can be incorporated into the modern system. The interviews were recorded using a digital recorder. In cases where participants were uncomfortable with electronic recording, extensive manual note-taking was performed to ensure data completeness while respecting participant preferences. In addition, self-guided walks and field observations were undertaken alongside interviews to visually identify therapeutic plants and specific herbal preparations mentioned by participants. Observational approaches supported interview data by placing participants' experiences in real-world contexts and verifying the described plants, materials, and locations.

### 2.6 Trustworthiness

In qualitative research, trustworthiness ensures that findings are accurate, reliable, and adhere to ethical considerations. In addition, it ensures that the research is credible and rigorous in addressing various reproductive health elements across different professionals (22, 23). In public health, trustworthiness is evaluated using several key elements, including credibility, dependability, confirmability, and authenticity (22). Table 1 lists the elements of trustworthiness and their relevance to this study.

**Table 1:** Elements of trustworthiness and their relevance.

	Elements of Trustworthiness	Aim	How trustworthiness was achieved	References
1	Confirmability	Ensure findings are shaped by participants experiences not researcher bias.	Data was thoroughly checked during data collection and analysis to ensure that the results are confirmed by other scholars. Comparing findings with existing literature on reproductive health and indigenous healing to ensure alignment and minimize personal bias. The research will be submitted to peer-reviewed journals.	(22)
2	Credibility	Truth of findings/internal validity	The results were discussed with experts to eliminate bias and ensure cross-checking of findings. The researcher spent sufficient time with respondents to gain a deeper understanding of traditional medicine insights.	(22)
3	Dependability	Ensure the research process is logical, traceable, and consistent.	The research was thoroughly reviewed and independently checked by the co-authors. Keeping consistent documentation of how traditional medicine-related data were collected, including cultural protocols, ethical considerations, and sensitivity regarding reproductive health topics	(24, 25)
4	Transferability	Applicability to other settings	The methodology used was ideal for ensuring that participants consented and were free to discuss sensitive issues related to reproductive health. A substantial number (41) of key informants were part of the research.	(23)
5	Authenticity	Present an honest, balanced representation of participant viewpoints.	The first author allowed participants to express different viewpoints regarding the services they offer in reproductive health. Allowing participants to express both positive and negative views about traditional remedies: perceived effectiveness vs. known risks. This was achieved by subscribing to research ethics.	(23)

### 2.7 Data analysis

Data gathered from traditional and health practitioners were transcribed verbatim, coded, and thematically analysed using MAXQDA Version 14. Codes from this qualitative software were then presented in the form of themes and subcategories, supported by direct quotes from the study respondents. Pictures of plants taken in the field were analysed using iNaturalist software, Google Images, and a botanist from the National University of Science and Technology to determine their families and species (classification).

## 3 Results

### 3.1 Demographic characteristics

Twelve of the twenty-six traditional practitioners were herbalists; all herbalists had ten or more years of experience in treating patients, and the ages of the traditional practitioners ranged from 33 to 72 (with an average of 49). Of the 15 health practitioners who participated in the study, two were medical doctors with less than ten years of experience. Health practitioner ages varied from 30 to 50 years (with an average of 40 years). Over 50% of both traditional and health practitioners were females. Table 2 summarises the participants' demographic characteristics.

**Table 2:** Demographic characteristics.

Traditional Practitioners (*n* = 26)							
Participant Number	Sex	Age	Marital Status	Work Description of TP/MP	Educational level	District	Experience (years)
1	Female	33	Single	Herbalist	Primary	Bulilima	13
2	Female	40	Married	Herbalist	Secondary	Bulilima	10
3	Male	78	Married	Bonesetters	Primary	Bulilima	20
4	Female	45	Married	Herbalist	Primary	Tsholotsho	11
5	Female	47	Married	Herbalist	Secondary	Bulilima	13
6	Female	25	Single	Herbalist	Secondary	Tsholotsho	10+
7	Male	45	Married	Herbalist	Primary	Tsholotsho	20+
8	Female	63	Widow	Diviners (Isangoma)	Primary	Tsholotsho	40+
9	Female	53	Divorced	Herbalist	Primary	Bulilima	30+
10	Female	59	Married	Diviners	Secondary	Bulilima	14
11	Female	67	Widow	Traditional Midwife	Secondary	Tsholotsho	30
12	Male	48	Married	Faith Healer	Tertiary	Bulilima	13
13	Female	40	Single	Herbalist	Secondary	Tsholotsho	15
14	Male	66	Married	Bonesetters	Tertiary	Tsholotsho	16
15	Male	45	Single	Diviners	Primary	Bulilima	14
16	Female	44	Single	Herbalist	Secondary	Tsholotsho	12
17	Female	42	Married	Herbalist	Secondary	Tsholotsho	12
18	Male	51	Married	Herbalist	Primary	Bulilima	11
19	Female	58	Married	Traditional Midwife	Secondary	Bulilima	10
20	Female	47	Married	Diviners	Secondary	Tsholotsho	13
21	Female	52	Single	Herbalist	Secondary	Tsholotsho	25+
22	Male	59	Married	Bonesetters	Primary	Bulilima	30+
23	Female	72	Widow	Diviners	Primary	Bulilima	30+
24	Female	53	Divorced	Traditional Midwife	Primary	Bulilima	20+
25	Male	46	Married	Herbalist	Primary	Bulilima	10+
26	Female	48	Married	Faith healer	Secondary	Tsholotsho	11+
Health Practitioners (*n* = 15)							
27	Female	44	Single	General Nurse	Tertiary	Bulilima	5
28	Female	37	Single	General Nurse	Tertiary	Tsholotsho	8
29	Male	38	Single	Medical Doctor	Tertiary	Bulilima	5
30	Female	45	Married	Nurse aid	Tertiary	Tsholotsho	7
31	Female	40	Married	General Nurse	Tertiary	Tsholotsho	9
32	Female	50	Single	General Nurse	Tertiary	Bulilima	9
33	Male	37	Single	Environmental Health Technician	Tertiary	Tsholotsho	7
34	Female	30	Single	General Nurse	Tertiary	Bulilima	5
35	Female	35	Married	Nurse Aid	Tertiary	Bulilima	8
36	Female	38	Married	General Nurse	Tertiary	Tsholotsho	7
37	Male	44	Single	General Nurse	Tertiary	Bulilima	8
38	Female	33	Single	General Nurse	Tertiary	Bulilima	4
39	Male	45	Married	Medical Doctor	Tertiary	Tsholotsho	6
40	Male	37	Divorced	General Nurse	Tertiary	Tsholotsho	7
41	Male	48	Married	Environmental Health Technician	Tertiary	Bulilima	9

### 3.2 Emerged themes from traditional and health practitioners

The collected and evaluated data revealed seven themes and twenty-two sub-themes. The themes associated with the use of traditional medicine in reproductive health among the Khoisan people of Bulilima and Tsholotsho included the types of traditional medicines, Administration and Preparation Techniques, Reproductive Practices, Knowledge Transmission, Perceived Efficacy and Safety, and Research and Education. A detailed overview of these themes, subcategories, and respondents' responses is presented in Table 3.

**Table 3:** Emerging themes from traditional and health practitioners.

	Themes	Subcategory	TP	HP	Supporting Quotes
1	Types of traditional medicines	(i) Plant-based, Animal-based	√	√	“*We use several plants to treat sexual and reproductive outcomes. In our San language, these include Mpakila, Mbandatshatsha, Khoriyabarwa, Gcamugcaba, and ibaso, Tatabadzimu, umgaranyeza, isiwubu*” (*Interviewee 23, Female-Diviner*)
					“I heard that grandmothers used to use bat feathers, burnt to ashes, and mixed with petroleum jelly is used to enlarge labia” (*Interviewee 27, Female-General Nurse*).
		(ii) Soil or Mineral-Based, Miscellaneous	√		“*Personally, I know some types of soils and other things that treat prostate cancer, but I cannot reveal them to you because our ancestors do not allow us*” (*Interviewee 3, Male- Born Setter*).
					“Snuff *is used to connect us with our ancestors, but it can be used to remove bad smell if put inside the vagina. For those who have several partners, one can put snuff after having sex so that the next person would not feel that her partner was having sex before*” (*Interviewee 14, Male-Born Setter*).
2	Administration and Preparation Techniques	(iii) Oral Administration	√		“*Sometimes for abortion they use black soot found in old kitchens and mix with warm water and taken orally, it can also be used in small quantities to treat stomach pains or menstrual pains, some say you can mix it with Coke or salt, but have never tried it*” (*Interviewee 11, Female- Traditional Midwife*).
					“A *combination of herbs commonly known as umvusankunzi is ground into powder, can be mixed with porridge, alcohol, mahewu, water, or tea, and taken orally among males. This herb cannot be mixed with milk”(Interviewee 18, Male- Herbalist)*
		(iv) Steam Baths	√		“*We use specific herbs to steam the patient's private parts and inhale while speaking healing words, so that they recover quickly*” (*Interviewee 15, Male-Diviner*).
					“I use *ulimi lwenkomo* (*Berkeya Setifera) barks soaked in warm water, steaming to tighten the vagina”* (*Interviewee 16, Female- Herbalist*).
		(v) Topical Application	√		“*One of the powerful plants used in the treatment of ulcers is ijoyi, which is normally found in front of kraals. You grind its thorns, burn them into ashes, and apply them to the genital ulcer. Within a few days or a week ulcer will be dry”* (*Interviewee 1, Female- Herbalist*).
3	Reproductive Practices/	(vi) Sexually Transmitted Diseases	√		“*I have treated several people who are suffering from idorobho (Gonorrhea), I was once also affected by drop and used sixteen different herbs mixed in water and taken as a decoction. Modern medicine does not cure STIs, but they make it to sleep” (Interviewee 18, Male- Herbalist)*.
					“*We do not use one herb in the treatment of STIs but a combination of more than five, which include boiled barks of Salvador*a *persica L (umtshekisane), Ximenia caffra (umthunduluka roots), Solanum campylacanthum (intume roots), Sclerocarya caffra (Marula/umganu barks), Drimia sanguinea (isagenama esibomvu), mix and drink to treat drops mostly” (Interviewee 20, Female-Diviner*).
					“*In most cases, I make sure that isanhlenhle is available in all mixtures as it is an important herb in the treatment of idorobho” (Interviewee 7, Male- Herbalist)*.
		(vii) Infertility and Menstrual Pains	√		“*There is a need that you mix goat dung, black soot from the kitchen, and salt, then boil and drink at least four to five cups a day to treat isilumo (menstrual pains*)” (*Interviewee 11, Female- Traditional Midwife*)
					“*We usually use barks and roots of isinama (Lerssetia Frutescens) mixed with boiling water to treat infertility both in males and females.” The roots are also boiled and used for bathing to remove bad omen (isidina/isinyama) that affects sexual appetite*.” *(Interviewee 9, Female- Herbalist*).
					“….*To reduce the number of menstrual days to normal, which is three, umviyo (Vangueria infausta) roots are mixed with water and taken orally; this mixture also works to treat infertility in females” (Interviewee 21, Female- Herbalist*).
		(viii) Cancer (Prostate and Cervical)	√		“*Several trees are used to treat cancer, and some are secret ingredients, we do not usually tell people. Gwava and sweet potato leaves boiled in water, then taken daily till healing, are used to treat prostate cancer*” *(Interviewee 13, Female- Herbalist*).
					“*Seeds, flowers, and leaves of inofi yephane (Agelanthus Pungu) are either mixed with water or taken as tea after grinding into powder to treat different types of cancer, especially cervical or prostate cancer” (Interviewee 3, Male- Herbalist)*.
					“As *much as Flacourtia indica (umqokolo) fruits are edible, they provide nutrients that boost immunity; its stem barks, when mixed with water and taken orally, are used to treat ulcers, genital warts, and gastro-related conditions*” *(Interviewee 5, Female- Herbalist*).
		(ix) Organ Enlargement or Tightening vagina	√	√	*“Normally, we use ivikani (Acacia arenaria) leaves, which are dried, ground into powder, and put in the vagina to tighten it. Some use igonde (Branchiostegal bragaei) barks soaked in water, and women squat on them”* (*Interviewee 24, Female- Traditional Midwife*).
					“…. *Chewing seven roots of Sida Acata for seven days increases the size of the male organ, especially if they also believe that it will enlarge*” (*Interviewee 8, Female-Diviner*).
		(x) Pregnancy	√	√	“*We have seen women who use donkey placenta and water snake/fish(inyeluka) to quicken the labour and delivery process in hospitals; they do not divulge the information unless there are complications*” (*Interviewee 39, Male-Medical doctor*).
					“*We use Pouzolzia hypoleuca (Isikhukhukhu) mixed with water and taken orally so that women in their last trimester deliver very fast*” (*Interviewee 14, Male-bone setter*).
4	Beliefs	(xi) Ancestral or spiritual	√		“*In most instances when treating patients with serious conditions, I had to consult my ancestors to know which herbs work well with your body. We do not just give people treatment without asking powers beyond us, just like if you go to a hospital and they scan you first*” (*Interviewee 20, Female-Diviners*).
					“*I usually dream of herbs either at night or during the day to treat diseases. I do not keep these medicines at home, but I go and harvest if there is a need. Some conditions do not only need herbs but prayers and intervention from God*” (*Interviewee 12, Male-Faith Healer*).
					“*There are also moments whereby ancestors and spirits refuse to treat someone but might refer to another person with better expertise or might even refer to the hospital*” (*Interviewee 26, Female-Faith Healer*).
		(xii) Cultural norms	√	√	“*We have tried and continue sensitising the San community to always visit the hospital for treatment of disease, but they lag and notify us that modern medicine makes them weak*, *they will continue following their culture of relying on herbs and hunting animals” (Interviewee 41, Male-Environmental Health Technician)*
					“*Long back we used not to wear clothes but animal skin, now we are losing our culture by believing these new things. I have never taken any tablet which makes me and my family strong, we try to preserve our culture by relying on natural things made by God”* (*Interviewee 23, Female-Diviners*)
					“*I have been HIV positive for the past five years but have never taken any tablets since I rely on herbs, which is our cultural belief. My wife and five kids tasted negative, showing that the herbs that I take are powerful”* (*Interviewee 14, male-Born-setter*)
5	Knowledge Transmission	(xiii) Inheritance and intergenerational	√	√	“*They usually share information among themselves, not with any other tribe, unless and only if they are now used to you, then they can trust you” (Interviewee 33, Male-Environmental Health Technician)*
					“*Knowledge of herbs and mixing of these concoctions runs in the family, not every San person knows herbs*” *(Interviewee 3, Male- Herbalist*
					“*We know each other in this community very well, we know who is best in treating different types of cancer, STIs, and other conditions as a result of witchcraft or even if you have lost your livestock*” (*Interviewee 20, Female-Diviners*).
		(xiv) Spiritual or ancestors’ revelation	√		*“Not everyone is chosen to be a healer; we are usually chosen by our ancestors, and denial of this gift might even make you sick for a long time. Failing to find a cure, there is a need for people to go for an apprenticeship (ukuyathwasa)” (Interviewee 22, Male- Born Setter)*
					“*The spirit talks to us in different ways, either in visions, dreams, or voice, notifying us to use a certain herb for a specific patient. Sometimes, we do not just use herbs without revelation from our ancestors. In some instances, you might be told to use a small baby who does not have sin to administer herbs for infertile people”* (*Interviewee 23, Female-Diviners*).
					“*As an African, there is no doubt that spirits do exist; there are some conditions we secretly advise patients to even seek traditional healing services, even though it is not recommended to do that” (Interviewee 23, General Nurse)*
		(xv) Apprenticeship	√	√	*“Just like any other school, traditional healers go for a year apprenticeship, they graduate in a ceremony where they choose a cow that was given by their family, and without prior knowledge of the apprentice, once they can choose that cow, then they will be trusted to treat the community” (Interviewee 37, Female General Nurse)*.
					“*Ukuthwasa is not an easy process as they are not treated well, sometimes not given food, bathing, and are usually given a lot of hard work, which is a strategy for them to know their purpose. Above all, when they are treated badly, the spirit mediums easily visit them during their dancing sessions”* (*Interviewee 8, Female-Diviner*).
6	Perceived Efficacy and Safety	(xvi) Patients and community validation	√	√	“*Modern medicines from hospitals do not cure STIs completely, but they make it to sleep, thereby recurring; there is a need to use herbs to destroy and kill the disease*” *(Interviewee 18, Male- Herbalist*).
					“*There are some herbs that are well known by almost everyone in the community, especially in their use and administration. I am sure that there is nobody who does not know isihaqa (Cassia abbreviate) and ivikani (Acacia arenaria) -even kids might tell you*” *(Interviewee 16, Female- Herbalist)*
					“*Patients come all the way from different countries, such as Botswana and South Africa, seeking our services. I have patients that I know who were cured from cancer, but I would not tell anyone who is not part of our family about our secret*” (*Interviewee 3, Male- Born Setter*).
					“…*those people are difficult to advise, so the best thing is to visit them and try to educate them since they trust their traditional way of managing health outcomes” (Interviewee 28, Female General Nurse)*.
					“*We have used these herbs for generation after generation, so indeed people trust our services. Additionally, we do not charge as much as other tribes that prioritize profit over all else. Our prices range from a bucket of maize, a goat to a bovine(s), sometimes free service” Interviewee 23, Female- Diviners)*
		(xvii) Infections		√	“*There is no doubt that majority of these topical prevalent cancers are also exacerbated by the utilisation of herbs especial those applied as topical or inserted in private parts*” *Interviewee 37, Male General Nurse)*.
					“*We have seen mothers who use herbs in managing umbilical and fontanelle in infants. That is a very sensitive area that should be treated with care; some even go to the extent of incising and putting some herbs on their forehead of their babies” Interviewee 34, Female General Nurse).*
		(xviii) Pain Relief	√		*“During the process of treatment, our herbs not only cure diseases but they relieve pain, for instance, Harpagophytum procumbens (umankunzane) acts as a pain relief in STIs” (Interviewee 18, Male- Herbalist)*.
					“*I do not doubt that after a few days given the traditional concoction on drop (STI), the pain will fade, especially when urinating, thereby giving relief during the treatment process, even though the herbs used do not taste good at all, they need a strong person who knows what he wants*” (Interviewee 18, Male- Herbalist).
		(xix) Falsification/Misuse by untrained users	√	√	“*I have a certificate to heal and treat people, those who are not registered or those without training from seniors end up cheating people, so patients have to be careful and ask the elders of the community before going for treatment*” *(Interviewee* 14 Male Born Setter).
					“*At the Hospital, we have seen cases where people have been erect for hours due to the use of herbs, and several miscarriages are occurring as a result of using traditional medicines to shorten labour. Let nature prevail and we avoid men's interference” (Interviewee* 39, Male-Medical Doctor)
					“*Never trust any drug that does not have a prescription and dosage. Surprisingly, these people might even give you an herb that is false. All I can say is sometimes healing starts in the mind. If believing that a certain herb will work, then there is a probability that indeed it will” Interviewee 36, Female General Nurse).*
		(xx) Risk profile	√		*“For the entire time treating people, there are no moments where my patients have severe outcomes after taking our concoctions. These medicines should be taken in correct doses to minimise risks. Matshisa is a very dangerous plant that should be used with great care” (Interviewee* 22 Male Born Setter).
					“*Everything comes from plants, even those tablets from the hospital need to be taken with caution. It is not easy to reverse damage that has been done by overdosing these herbs, for treating males who overdosed on herbs to boost, the only way is to put a cold, moist towel around the organ”* (Interviewee 25, Male- Herbalist).
		(xxi) Availability	√		*“Some of the herbs that we use are not already available but are found across countries, some are found in Nyamandlovu, or sometimes we could travel as far as Botswana in search of these medicines, especially if directed by the vision” (Interviewee* 14 Male Born Setter).
					“*Not every plant can be stored for a long period of time; some end up being poisonous, so we treat based on patient needs rather than storing them. We just store basic plants” (Interviewee 3, Male- Herbalist)*.
					“*We are also affected by seasons as some herbs are available only during the rainy season, but luckily we stay close to the Manzamnyama river, which is our number one source for herbs” (Interviewee 16, Female- Herbalist)*
7	Research and Education	(xxii) Scientific Collaboration	√	√	*“There should be a database of these common herbs that are used by people and methods of managing them. With technology, it is better to have mobile applications with a list of traditional herbs. A lot needs to be researched in this field, and we know even poisonous plants” (Interviewee 41, Male-Environmental Health Technician)*
					“*Hospitals should be a one-stop center that gives patients satisfaction according to their preferences. Traditional healers should have their corner in the Hospital such that nurses can refer patients to us and vice versa”* (Interviewee 25, Male- Herbalist).
					“*Without information and knowledge transmission, we will always remain outdated in the medical field. There is a need for different experts in medicine to conduct periodic workshops sharing information that is crucial for our patients. Priority should be given to the health of our patients” (Interviewee 39, Male-Medical Doctor)*.
					“*I do not know more about herbs, but I think these traditional medicines should be part of the school curriculum starting from primary, secondary up to tertiary level so that these plants remain known not only to experts but to everyone” Interviewee 35, Female-Nurse aid).*
		(xxiii) Counselling and Health Promotion		√	*“We do ask patients history to determine predisposing factors and exposure to these herbal medicine before commencement of treatment, health education is given to patients and caregivers on the dangers of using herbs in conjunction with ARVs” (Interviewee 29, Male-Medical Doctor)*
					“*It is not easy for people to accept their HIV status, majority stay in denial stage for long period of time and in most cases, they will be using concoctions in treating HIV. We do make follow ups and counsel such patients as they are at risk of committing suicide in such of viable treatment for advanced HIV” (Interviewee 34, Female General Nurse).*

#### 3.2.1 Types of traditional medicines

Two subcategories were derived from the above-stated theme: plant- or animal-based, and soil- or mineral-based, with a miscellaneous category. Both traditional medicine and health practitioners are indicated that medicines are derived from either plants or animals. The Khoisan people have stated that several plants are used in the management of STIs in their local languages. Health practitioners have reported that some patients use bat feathers mixed with oil or petroleum jelly to elongate the labia which is believed to enhance sexual excitement. In addition, Traditional Practitioners (TPs) noted that an STI commonly known as a drop (gonorrhea) is not treated with only one type of herb but with more than five herbs, and one healer even stated it needs 16 herbs in order to cure it. Roots have been cited as the primary part of the plant used to treat various ailments. In addition, traditional practitioners have indicated that prostate cancer can be cured using a type of soil combined with secret ingredients. Detailed information on the types of traditional medicines is provided in Table 3.

#### 3.2.2 Administration and preparation techniques

Three subcategories emerged from this theme: Oral Administration, Steam Baths, and Topical Application. Traditional practitioners have noted that most herbs are dried and ground into a powder, mixed with porridge, tea, water, or mahewu, and taken orally. Some herbs were stated to be used by applying them directly to ulcers, while others were used as steam. Participants' direct naractives on the themes of administration and preparation techniques are presented in Table 3.

#### 3.2.3 Reproductive practices

Five subcategories emerged from this theme: sexually transmitted diseases, infertility, menstrual pain, cancer, organ enlargement or tightening of the vagina, and pregnancy. Respondents cited several herbs for sexually transmitted diseases, especially drops, and management of menstrual pain or heavy flows, as revealed by traditional practitioners. They also reported that there are herbs that are used to tighten the vagina, enlarge the male organ, or make the body hot during sexual intercourse. *The TPs stated Agelanthus Pungu and Lerssetia Frutescen*s as herbs that treat cancer and infertility, respectively. Reproductive outcomes and their five subcategories are listed in Table 3.

#### 3.2.4 Knowledge transmission

Inheritance, intergenerational, spiritual or ancestral revelation, and apprenticeship were subcategories derived from the theme mentioned above. Informants noted that knowledge of herbs was passed down through the bloodlines of their families. TPs cited that ancestors do not choose them to perform healing practices, as they reveal different types of herbs. They also noted that those chosen for an apprenticeship were trained for more than a year to cure several diseases. Respondents' quotes on how traditional knowledge was transmitted are highlighted in Table 3.

#### 3.2.5 Perceived efficacy and safety

Six subcategories were derived from the themes: patient and community validation, Infections, Pain Relief, Falsification/Misuse by untrained users, Availability, and Risk profile. Key informants highlighted that modern medicines do not cure STIs completely, but they suppress them (make it sleep), which will cause the disease to recur; hence, the use of herbs destroys it. They also noted that patients come from different countries seeking their services; hence, they have witnessed those who were once cured of cancer. In contrast, health practitioners have cited that these herbs can cause infections among infants during the management of the fontanelle and umbilical cord, even though TPs note that their herbs have low-risk profiles. The respondents indicated that patients should be alert to the risk of being given the wrong prescription by untrained healers or those without proper knowledge. The perceived efficacy themes and safety are presented in Table 3.

#### 3.2.6 Beliefs

Ancestral, spiritual, and cultural norms were the subcategories extracted from beliefs. TPs highlighted that they do not only treat people without seeking guidance from beyond, and there are times when ancestors refuse to treat someone; thus, we refer the patient to someone else. The San community subscribes to herbal medicines because they believe that their use will not divert from their culture, thereby preserving it. The direct quotes on participants' beliefs are presented in Table 3.

#### 3.2.7 Research and education

Two subcategories came from research and education: scientific collaboration, Counselling, and health promotion. One nurse noted that these traditional medicines should be included in the school curriculum for students so that they are recognised by everyone, not just a specific community. Respondents concur that they should work together once the information is available through workshops. TPs noted that they should be given space in the hospital so that they can practice their services in open and accessible places. One nurse stated that counselling services and health education should be prioritised mostly for HIV + patients, as they might stay in the denial stage for a long period of time. Table 3 presents the themes related to research and education.

### 3.3 Traditional medicines used in reproductive health

The informants mentioned several traditional medicines used in the management of reproductive health. They highlighted the methods of preparation, administration, and health conditions being treated. The most commonly stated herbs belong to the Fabaceae family. Table 4 shows Traditional Medicines used in STIs and STDs, Table 5 shows Traditional Medicines used in Sexual Practices, and Table 6 highlights Traditional Medicine utilisation in maternity.

**Table 4:** Traditional medicines used in STDs.

	Family	Scientific Name	Local Name	TM Type	Part Used	Preparation and administration technique	Disease Treated
1			Umchithazulu	Plant	Roots	Mix with water and drink till cured	Gonorrhoea
2	Hyacinthaceae	*Drimia sanguinea*	Isagenama Esibomvu (Red squil)	Plant	Tuber	Chop into small pieces and soak in water, then drink	Gonorrhoea
3			Mbojana	Plant	Roots	Mix with water and take as a decoction	Gonorrhoea
4	Polygalaceae	*Securidaca longepedunculata*	Mfumfu/Itshabela (Violet tree)	Plant	Roots	Put in water, but in a small quantity, as it is very strong and drink	Gonorrhoea
5	Papaveraceae	*Argemone mexicana L*	Umthwentwe (Prickly poppy)	Plant	Roots	Grind roots into powder, mix with water, and take orally	Prostate cancer, uterus cleaning, Gonorrhoea
6	Olacaceae	Ximenia rogersii/*Ximenia americana L*	Umswanja (Blue Sour Plum)	Plant	Roots	Mix with water and take orally till healed.	Gonorrhoea
7	Olacaceae	*Ximenia caffra*	Mthunduluka obomvu (Sour Plum)	Plant	Roots	Mix with water and drink daily	Drop (idorobho)
8	Musaceae	*Musa acuminata, Musa balbisiana*	Banana	Plant	Roots	Mix with water and drink	Drop
9	Fabaceae	*Acacia natalitia*	isiringa (Acacia karroo)	Plant	Roots	Mix with water and take orally	Drop
10			Mpepe	Plant	Roots	Mix with water and take orally	Drop
11	Ranunculaceae	*Clematis villosa/Clematis stanleyi*	Umatshisa (Fire-and-Ice plant)	Plant	Roots	Mix with water in a very small quantity	Drop
12	Fabaceae	*Elephantorrhiza elephantin*	Ntolwane Enkulu (elephant's root)	Plant	Roots	Mix with water and taken as a decoction	Drop
13	Fabaceae	*Elephantorrhiza burkei Benth*	Ntolwane Encane (Broad-pod elephant-root)	Plant	Tuber	Mix with water and drink	Drop
14	Fabaceae	*Colophospermum mopane*	Mopane	Plant	Roots	Grind into powder and mix with water, then take orally.	Drop
15	Asphodelaceae	*Aloe Maculate, Aloe saponaria*	Icena	Plant	Leaves	Mix with water	Drop
16	Salvadoraceae	*Salvadora persica L*	umtshekisane	Plant	Roots	Mix with water and drink	Drop
17	Solanaceae	*Solanum campylacanthum ‘incanum type*	Intume enkulu	Plant	Roots	Mix with water and drink	Drop
18			ijoyi	Plant	Thorns	Dry the plant, grind and put the powder on the ulcer	Genital ulcers
19			Nkamamasane Enkulu	Plant	Roots	Put it with other STIs plants as a neutraliser, or act as a sweetener	
20	Anacardiaceae	*Sclerocarya caffra*	Umganu (Marula)	Plant	Barks	Boil and Drink	HIV
21			Mkombegwa	Plant	Barks	Boil and Drink	Drop
22	Fabaceae	*Brachystegia bragaei*	Gonde	Plant	Roots	Mix with water and drink	STIs
23			Isanhlenhle	Plant	Roots	Mix with water and drink	STIs
24	Asphodelaceae	*Haworthiopsis attenuata*	Intelezi (Zebra Plant)	Plant	Roots	Mix with water and drink	STIs
25	Fabaceae	*Acacia galpinii*	Umkhaya (Monkey thorn)	Plant	Roots	Mix with water and drink	STIs
26	Fabaceae	*Cassia abbreviate*	Isihaqa (Long-tail cassia)	Plant	Roots	Mix with lemon roots in water and drink	HIV and stomach pains
27	Fabaceae	*Colophospermum mopane*	Mopane	Plant	Bark	Mix with umvagazi barks and drink	HIV
28			Khalimela	Plant	Roots	Mix with water and drink	HIV
29	Burseraceae	*Commiphora glandulosa*	Iminyela/Unqobho obomvu	Plant	Tuber	Eat it raw	Boost immunity in HIV patients
30	Combretaceae	*Combretum imberbe*	Umtswiri	Plant	Roots	Mix with water and taken orally	Strengthening of bones in HIV patients
31	Euphorbiaceae	*Jatropha curcas*	Jatropha	Plant	leaves	Incise with a razor and put on an ulcer	Ulcers
32	Papaveraceae	*Argemone mexicana*	Mexican Popy Plant	Plant	Roots	Roots help with constipation, and fluid when a plant is injured can cure warts or bruises	Cancer,Genital warts
33	Loranthaceae	*Agelanthus Pungu*	Inofi Yephane	Plant	Whole Plant	Can be taken as tea, or dry the plant, then grind into powder and mix with porridge, and taken daily	Cancer
34			Umhlambamanzi	Plant	Roots	Boil and drink	All STDs
35			Umanana/umqalothi	Plant	Roots and barks	Boil and Drink	All STDs, especially one with ulcers
36	Pedaliaceae	*Harpagophytum procumbens*	Umankunzane (Devil's Claw)	Plant	Leaves, Flowers, Roots	Boil and drink	STIs, HIV, and pain relief
37	Convolvulaceae	*Ipomoea batatas L*	Imbambayila (Sweet potato)	Plant	Leaves	Boil leaves and drink	Treat prostate cancer
38	Cactaceae	*Opuntia ficus-indica*	Dorofiya (Prickly Pear cactus)	Plant	Bark	Peel and soak in water overnight, and take as a decoction	Boost immunity among HIV
39	Hypoxidaceae	*Hypoxis hemerocallidea*.	Izambane (wild Potato)	Plant	Tuber	Put in boiling water and drink	Boost CD4 in HIV people
40	Salicacea	*Flacourtia indica or Dovyalis caffra*	Umqokolo	Plant	Stem Bark	Mix with water and drink	Treat cancer, gastrointestinal diseases, and ulcers
41	Myrtaceae	*Guaiava pyriformis and Guajava pumila.*	Gwava	Plant	Leaves	Boil and drink daily	Boost the immune system for Prostate cancer
42	Myrtaceae	*Psidium guajava L*	Gwava Leganga	Plant	Roots	Roots mix in water	Helps cure skin herpes
43			Muzeze	Plant	Seeds	Put in water and drink for at least one week	Genital warts
44	Fabaceae	*Dichrostachys cinerea*	Ugagu (Sickle bush)	Plant	Roots	Mix with water and drink daily	Treat cancer
45	Lamiaceae	*Leucas martinicensis/Leonotis ocymifolia*	Uluju lwenyoni	Plant	Stem bark, Leaves	Mix with water and taken orally. Leaves are applied topically to treat sores	Treat cancer

**Table 5:** Traditional medicines used in sexual practices.

	Family/Specie	Scientific Name	Local name	TM Type	Part Used	Preparation and administration technique	Purpose of use
1	Combretaceae	*Acacia arenaria.*	Ivikani (Sand Thorn)	Plant	Leaves	Dried and put in the vagina	Tighten vagina
2			Umgugudu	Plant	Barks	Dried and put in the vagina	Tighten vagina
3	Pteropodidae	*Chiroptera spp*	Bat (umanlembe)	Animal	Feathers	Burn and powder mixed with petroleum jelly or other oils, and massaging the labia	To enlarge the labia
4	Fabaceae	*Brachystegia bragaei*	Gonde	Plant	Barks	Put it in water, then squat	Tighten vagina
5	Rubiaceae	*Vangueria infausta*	Umviyo (Velvet Wild Medlar)	Plant	Roots	Roots and drink	To cut the number of menstruation days, infertility
6			Mchithazulu	Plant	Roots	Dry and mix with water, then drink	Reduce menstruation days
7			Inokonoko (Goat dung)	Animal	dung	Mix with isinyayi, salt, then boil and drink 4–5 cups per day	Isilumo/menstrual pains
8			Isinyayi (Black soot from thatched huts)			Mix with water and drink early in the morning	Stomach pains during menstruation
9	Fabaceae	*Pterocarpus angolensis*	Umvagazi (African Teak/wild teak	Plant	Barks	Mix with water and drink	Menstrual pains
10	Fabaceae	*Peltophorum africanum.*	Umsehla (African wattle)	Plant	Barks	Mix with water and drink	Menstrual pains
11	Fabaceae	*Brachystegia boehmii.*	Itshabela	Plant	Barks	Mix with water and drink	Menstrual pains
12			Isikhukhula (Flooded River shrubs)	Shrubs		Mix with water and drink	Infertility
14	Gnetum	*Gnetum africanum*	Umgoma (African jointfir)	Plant	Fruits, leaves	Mix the fruit with water and drink	Infertility, warts, and cancer
15			Snuff			Mix with Coke and bathe your vagina.	To tighten the vagina and remove odour
16	Araliaceae	*Cussonia Panuculata*	Msenge	Plant	Roots	Mix with milk and drink, or eat raw at least 3 times a day	Makes the male body hot and improves back born
17	Asphodelaceae	*Aloe Maculate, Aloe saponaria*	Icena	Plant	Barks	Grind, mix with cold water, Drink morning and evening	Improves male and female strength
18	Asphodelaceae	Aloe Cooper	Icacane	Plant	Roots	Boil in water and drink	Menstrual pains or isilumo
19	Asphodelaceae	*Aloe vera*	Inkalane	Plant	Barks	Dry and burn the bark into ashes. Put it in soup or porridge: Eat for three days	Reduce Heavy menstrual flow
20	Asteraceae	*Berkeya Setifera*	Ulimi lwenkomo (Buffalo tongue)	Plant	-Roots	-Boil and drink	-All sexual diseases and heavy menstrual flow in females
					-barks	-Put in warm water and steam the vagina	-tighten the vagina
21	Anacardiaceae	*Harperphylum Caffrum*	(Wild plum)	Plant	Bark	-Boil and drink, Bathing	-Infertility or treat miscarriages
						-boil and drink	-improve sexual appetite for both male and female, all STDs
						-boil and drink so that you vomit	-for a male who slept with someone's wife
22	Apocynaceae	*Rauvolfia caffra,*	Umhlambamanzi (quinine tree)	Plant	Bark	Boil the drink and bath the vagina	Remove water from the vagina
23	Fabaceae	*Lerssetia Frutescens*	Isinama (sutherlandia/ cancer bush)	Plant	-Bark and Roots	Boil and Drink	-infertility for both male and female, cancer
					-roots		-reduce heavy menstruation
					-roots	-boil and bath	-remove bad omen during sex (isidina/isinyama)
24	Pedaliaceae	*Harpagophytum procumbens.*	Umankunzane (Devil's Claw)	Plant	Thorns	Dry, burn powder and eat	Boost sexual appetite in males
25	Fabaceae	*Pterocarpus angolensis*	Umvagasi	Plant	Leaves and fruit	Grind into powder and burn, then drink	Boost male sexual appetite and last long in bed
						Apply powder on the male organ	
26	Fabaceae	*Acacia nilotica*	Isanqawe/Umtshatshatsha (Umbrella thorn)	Plant	Seeds	Dry and make into powder	Ulcers, bad discharge, or vagina odour
27	Malvaceae	*Sida Acata*	Wire weed	Plant	Roots	Chewing 7roots for 7 days	Increase manhood size and boost immunity
28	Lamiaceae	*Leonotis leonurus*	Lion tale (Ibetshulebadala)	Plant	Leaves and roots	Make tea and drink	Fibroids and menstrual discomfort
29	Lauraceae	*Persea americana*	Avocado pear (Mukotapeya)	Plant	Seeds	Dry and grind, put in porridge, but don’t overdose as it is poisonous	Boost Sexuality, Cancer, and Immunity

**Table 6:** Traditional medicine utilisation in pregnancy.

	Family	Scientific Name	Local name	TM Type	Part Used	Preparation and administration technique	Condition
1	Fabaceae	*Brachystegia bragaei*	Impande yegonde	Plant	Leaves	Peel and mix with water, then take orally after delivery	To clean the system
2			Isanhlenhle	Plant	Roots	Mix with water and taken orally	To clean the uterus after delivery and treat other diseases
3			Inqwatshi yedonki (Donkey Placenta)	Animal	Placenta	Mix with water and drink	Fast delivery
4			Mfutho	Plant	Roots	Taken orally	Last trimester to quicken delivery
5	Acanthaceae	*Hypoestes aristata*	Idolo lenkonyane	Plant	Plant	Mix with mvagazi and take orally	Reduce heartburn and vomiting during early stages of pregnancy.
6			Inyeluka (water snake/fish)	Animal	Skin	Mix with water and drink	to shorten labour and ease delivery
7			Elephant Dung	Animal	dung	Burn and steam vagina	To stop bleeding during Pregnancy
8			Isinyayi (soot from old, thatched kitchen)			Mix with water and drink, or Coke and salt	Fast delivery and abortion
9	Malvaceae	*Adansonia digitata*	Umkhomo (Baobab)	Plant	Roots	Grind into powder, bathing and drinking	Stabilise Pregnancy
10	Fabaceae	*Colophospermum mopane*	Iphane (Mopane)	Plant	Roots	Grind and drink	Fast delivery
11	Urticaceae	*Pouzolzia hypoleuca*	Isikhukhukhu (Snuggle-leaf)	Plant	Whole plant	Mix with water and drink	Fast delivery
12	Anacardiaceae	*Sclerocarya caffra/ birrea*	Umganu (Marula)	Plant	Barks	Put in water and drink	After birth to clean the stomach

## 4 Discussion

### 4.1 Demographic characteristics

The study found that most service providers were herbalists with more than ten years of experience treating reproductive health ailments. This finding is consistent with a study conducted in Bulilima, which indicated that traditional practitioners have more than ten years of experience (15). A different perspective is offered by a study conducted by Kadir et al. (26), who noted that the experience of traditional practitioners is less than ten years (26). In the modern health sector, research has noted that there are fewer medical doctors than general nurses. These findings align with the literature, which indicates a low number of doctors in rural areas of developing countries due to poor infrastructure (27). In contrast, medical doctors are more common in high-income rural areas than in urban areas (28). Additionally, the research revealed that the average age of traditional practitioners was higher than that of health care practitioners. These findings concur with those of a study conducted in Zimbabwe, which reported a mean age of 55 years for traditional healers (29).

### 4.2 Sexual practices

The results revealed that a combination of herbs commonly known as umvusankunzi can be mixed with alcohol, mahewu, water, tea, and porridge and taken orally to boost sexual appetite, but cannot be mixed with milk. A study conducted in South Africa noted that a mixture of different herbs was used to boost libido in both men and women (30). Furthermore, Akramjanovna 2024 supports our study by denoting that milk is not used in herbal medicine because it is not a good solvent (31). *Lerssetia Frutescens* (Isinama) mixed with boiling water has been shown to treat infertility in both males and females, as well as enhance sexual appetite. Studies on *Lerssetia frutescens* have linked it to its use in cancer, blood purification, and anti-stress, in addition to infertility (32). Currently, *Lerssetia Frutescens* can not be recommended as a cancer control since it lack proper selectivity controls, realistic dosages, and human clinical validation and is not a direct treatment to infertility. The plant have unique amino acids, triterpenoid glycosides, flavonoids, and pinitol but cancer remains experimental and infertility treatment claims are not supported by evidence (33).

*Acacia arenaria* (ivikani) leaves, which are dried and ground into powder, and igonde *Brachystegia bragaei* (igonde) barks are soaked in water, and women squat on them to tighten the vagina. The phytochemical properties of other species within the same family, such as *Brachystegia eurycoma* and *Acacia sp*., differ from those found in this study (34, 35). A mixture of goat dung, black soot from an old, thatched kitchen, and salt was boiled and taken orally at least four–five cups a day to treat menstrual pain. Chewing *Sida cata* roots are believed to enlarge male organs. This is supported by the literature that highlights *that Sida acata* can be used for testicle enlargement (Pimple et al.) (36). In addition, an experimental study conducted in rats indicated that it has hormonal effects and can induce pregnancy at higher doses but are no scientific, clinical, or pharmacological studies showing increased testicular volume, stimulation of spermatogenic tissue growth and increased Leydig cell mass in humans (37).

### 4.3 Sexual transmitted diseasesand cancer

Herbalists and diviners have indicated that they use more than one herb in the treatment of STIs, particularly gonorrhea, which is commonly known as idorobho. They stated that a combination of more than five, including boiled bark of *Salvadora persica L* (umtshekisane), *Ximenia caffra* (umthunduluka roots), *Solanum campylacanthum* (intume roots), *Sclerocarya caffra* (Marula/umganu barks), and *Drimia sanguinea* (isagenama esibomvu), are mixed and consumed to treat drops. These plants are supported by several authors who have indicated their use in treating a wide range of venereal diseases, including STDs (38–40). Furthermore, *Solanum campylacanthum* roots, *Ximenia caffra* roots and *Drimia sanguinea* posses phytochemical consituents such as tanins, flavanoids and phenolic acids show inhibitory effects against Gram-negative bacteria such as *Neisseria gonorrhoeae* (41). In contrary, Drimia has been deemed unsafe to use under uncontrolled environments (42).

The results indicated that the seeds, flowers, and leaves of *Agelanthus Pungu* (inofi yephane) are mixed with water or taken as tea to treat various types of cancer, particularly cervical or prostate cancer. A more plausible laboratory explanation is given by a study conducted by Mlilo S (2025), who also indicates that *Agelanthus Pungu* possesses phytochemical properties in the treatment of cancer (43). Roots of *Dichrostachys cinerea* (ugagu), the whole plant of *Leucas martinicensis* (uluju lwenyoni), *Guaiava pyriformis*, and *Guajava pumila* leaves have been used to treat cancer. A study by Magbool (2025) highlighted that *Dichrostachys cinerea* is a medicinal plant with potential therapeutic uses, including the treatment of cancer, because it contains alkaloids (44). Additionally, it was noted that *Leucas martinicensis* possesses chemical characteristics that target and combat cancer cells (43). Researchers have deemed guava leaves a promising plant for combating breast and prostate cancers as they have high concentrations of quercetin and gallic acid which blocks cancer-promoting cytokines and uncontrolled cell division (45–47). It was also noted that *Flacourtia indica* (umqokolo) bark, when mixed with water and taken orally, is used to treat ulcers, genital warts, and gastro-related conditions. Similarly, owing to their inflammatory properties, *Flacourtia indica* roots and bark have been shown in the literature for use in the treatment of various health conditions, including gastric ulcers, venereal diseases, and infertility (43, 48).

Nurses indicated that they discouraged the use of traditional medicines while taking ARVs, while traditional practitioners indicated that they used herbs during HIV management. This is further supported by various scholars who indicate that health professionals have negative viewpoints, whereas traditional practitioners support the use of herbal medicine among HIV-positive patients (49–51). Researchers have also found that utilising traditional medicines while taking ARVs reduces adherence to ART (Trevor and Jane) (52).

### 4.4 Maternal health

*Colophospermum mopane* (Iphane), *Pouzolzia hypoleuca* (Isikhukhukhu), water snake/fish (Inyeluka), soot from an old, thatched kitchen (Isinyayi), and Donkey Placenta were used to shorten labor and ease of delivery. The study results support and concur with those of a study conducted in Zimbabwe on maternal health and traditional medicines (15). Colophospermum mopane compounds, including flavonoids (quercetin, kaempferol), tannins, and saponins, are thought to help control gastrointestinal disorders, minimize infection risks, and offer important nutrients to pregnant woman (53). Soot from old, thatched kitchens have been used for abortion in the early stages of pregnancy and for quick delivery during labor in the last trimester. No information was found on online search engines regarding soot use during pregnancy.

### 4.5 Privacy and confidentiality

Some participants only revealed common herbs without indicating the secret ingredients that treat STIs and prostate cancer. Studies have indicated that disclosure of only commonly known herbs reflects traditional practitioners' efforts to safeguard the intellectual property rights of native medicinal knowledge, as specific ingredients and formulations for treating STIs and prostate cancer are considered proprietary and culturally protected knowledge (54, 55). It has been noted that standardization and collaboration of THP with modern systems are challenging, as they do not disclose information on some of the herbs they use (50, 56). Furthermore, current regulations, such as the Traditional Medical Practitioners Act of 1981, the National Health Strategy (2016–2020), and the Medicines Control Authority of Zimbabwe, primarily focus on the usage and standardization of traditional medicine rather than its incorporation into the modern system (57). The study noted that diviners consulted their ancestors to determine which herbs to use when treating various reproductive diseases. This is supported by Qwabi et al. (58), who indicated that the healing and mixing of herbs requires strength from the above powers, which include ancestors (58).

### 4.6 Safety and availability

This research also revealed that mobile applications with a list of traditional herbs are needed to distinguish between useful and poisonous plants. The recommendation to develop mobile applications listing traditional herbs poses dangers of knowledge commodification, as digitising such material may allow for the extraction and commercialisation of indigenous knowledge without proper community governance, cultural context, or benefit sharing (59). The literature reveals that knowledge of traditional plants, especially those that are poisonous, should be accumulated and made known to the public but does not indicate the need to merge with current technologies (60, 61). The results indicated that hospitals should be a one-stop center, allowing traditional healers to have a designated space within the hospital so that nurses can refer patients to them and vice versa. Scholars support the notion of one health concept, whereby all experts are housed in one central location to address different health conditions, especially in rural areas of developing countries, where coverage is limited (62). Although the One Health approach promotes collaboration, aggregating all experts into a single system may exacerbate power imbalances, as biomedical institutions frequently dominate governance and standards, potentially marginalizing traditional health practitioners and their knowledge systems in rural areas (63).

## 5 Limitations

This study was part of a larger study funded by the National University of Science and Technology Research Board and was conducted on a limited budget. Therefore, we did not have funds to compensate the participants for their time in participating in the study, leading to some of them being frustrated to participate in the study. Most Khoisan people needed a token of appreciation for their services, in the form of alcohol, food, and clothes. Some traditional practitioners do not disclose their secret key ingredients and only state those that are widely known by the community. Some herbs were not available during the study period, as they were harvested from different parts of the country and, in some instances, outside the country. Some people do not keep herbs as they use them when needed, thereby making it difficult to identify them. The reliance on self-reported data from traditional practitioners raises the prospect of social desirability bias, as participants' responses may have been altered to match perceived expectations or cultural norms. Some herbalists intentionally concealed crucial constituents from their medicines, disclosing only well-known herbs, which may have resulted in insufficient documentation of medical formulas. The findings were not backed by laboratory-based phytochemical or pharmacological validation, limiting our capacity to scientifically establish the efficacy, safety, and bioactive qualities of the reported herbal remedies.

## 6 Conclusion

Undoubtedly, the Khoisan people of Zimbabwe rely heavily on traditional medicine to treat sexually transmitted diseases, control pregnancy, and engage in sexual behaviors. Most herbal treatments are derived from plant roots and provided to patients as decoctions. Traditional practitioners have classified STIs into two categories: *dorobhos* (drop) and ulcers. Healthcare workers should have access to a database or mobile application containing information on common plants used in reproductive health as well as knowledge of toxic herbs. These herbs should be included in the school curricula so that they are well known among all age groups. An ethnographic study should be conducted to determine the hidden constituents that are not limited to common herbs. An ethnographic study should be conducted to determine the hidden constituents that are not limited to common herbs. There is also a need for follow-up studies on patients with cancer who have been cured by Khoisan healers.

## Data Availability

The raw data supporting the conclusions of this article will be made available by the authors, without undue reservation.
